# Effect of Biochanin A on Serum Visfatin Level of Streptozocin-Induced Diabetic Rats

**DOI:** 10.5812/ircmj.15424

**Published:** 2014-09-05

**Authors:** Reza Azizi, Mohamad Taghi Goodarzi, Zahra Salemi

**Affiliations:** 1 Department of Biochemistry, Arak University of Medical Sciences, Arak, IR Iran; 2 Molecular Medicine Research Center, Hamadan University of Medical Sciences, Hamadan, IR Iran; 3 Molecular and Medicine Research Center, Arak University of Medical Sciences, Arak, IR Iran

**Keywords:** Visfatin, Biochanin A, Diabetic Rats

## Abstract

**Background:**

Bioflavonoids are well known for their multi directional biologic activity including antidiabetic effect. It has been demonstrated that flavonoids can act as insulin secretagogue or insulin mimetic agents.

**Objectives:**

This experimental study was designed in Arak University of Medical Sciences, Arak, Iran, to investigate the effects of biochanin A (a bioflavonoid) on fasting blood glucose (FBG), body weight, glycosylated hemoglobin (HbA_1_c), lipid profile, serum enzymes, and visfatin of streptozocin-induced diabetic rats.

**Patients and Methods:**

We used 24 male Wistar rats and randomly allocated them to four groups of six rats. One group was randomly assigned as control and diabetes was induced in three other groups by administration of streptozocin (35 mg/kg of body weight) intraperitoneally. The groups received the following treatments: group 1 (control), 5% DMSO; group 2 (diabetic control), 0.5% DMSO; and group 3 and 4, respectively 10 and 15 mg/kg biochanin A for 30 days. Body weight and biochemical parameters including FBG, HbA_1_c, lipid profile, aspartate aminotransferase (AST), alanine aminotransferase (ALT), alkaline phosphatase (ALP), and visfatin were measured in all rats.

**Results:**

FBG level was significantly reduced in treated diabetic rats (139.8 ± 9.3 and 206 ± 11 mg/dL in groups 3 and 4, respectively) in comparison to the diabetic control (295.1 ± 14 mg/dL) (P < 0.05). Administration of biochanin A significantly decreased HbA_1_c in group 3 (6.66 ± 0.33) and group 4 (7.11 ± 0.31) in comparison to the diabetic control group (8.26 ± 0.44) (P < 0.05). Levels of serum visfatin were improved to near normal levels in the treated rats (249 ± 35.5 and 161.33 ± 13.07 in groups 3 and 4, respectively) in comparison to the diabetic control (302.17 ± 19.4) (P < 0.05). Furthermore, biochanin A showed a protective effect against weight loss in diabetic rats (P < 0.05). In treated rats, serum total cholesterol, triglyceride, and low-density lipoprotein cholesterol (LDL-c) were significantly decreased and high-density lipoprotein (HDL-c) was increased in comparison with the diabetic control group. In addition, biochanin A restored the altered plasma enzymes (AST, ALT, and ALP) activities to near normal. Histopathologic examination of the pancreas also indicated that biochanin A had protective effects on β-cells in streptozocin-induced diabetic rats.

**Conclusions:**

This study demonstrated that biochanin A possessed hypoglycemic and antilipemic activities and could increase visfatin expression, which suggests its beneficial effect in the treatment of diabetes.

## 1. Background

Diabetes mellitus is a metabolic disorder with the origins as old as mankind and its incidence is considered to be high worldwide (1). It is characterized by hyperglycemia resulting from defects in insulin secretion, insulin action, or both. The long-term manifestation of diabetes can result in the development of some microvascular complications including neuropathy, nephropathy, and retinopathy and macrovascular complications including heart disease, stroke, and peripheral vascular disease, which can lead to ulcers, gangrene, and amputation (2). Even though the currently available drugs (insulin, sulfonylureas, biguanides, and thiazolidinediones) may be valuable in the management of diabetes, these drugs are usually accompanied by considerable side effects such as hypoglycemia, drug resistance, edema, and weight gain (3). Several species of plants have demonstrated potential for treatment of diabetes with lesser or no side effects (4). It is reported that about 800 plants may possess antidiabetic properties (5). Bioflavonoids are well known for their multidirectional biologic activities including antidiabetic effects. Numerous studies have already demonstrated the hypoglycemic effects of flavonoids in different experimental models and treatments. Flavonoids can act per se as insulin secretagogue or insulin mimetic agents, probably by influencing the pleiotropic mechanisms, to attenuate the diabetic complications. Flavonoids have been found to stimulate glucose uptake in peripheral tissues and regulate the activity and/or expression of the rate limiting enzymes involved in carbohydrate metabolism pathway (6). Biochanin A, an isoflavone in the class of phytochemicals existing in red clover, cabbage, and alfalfa, has an inhibitory effect on benzo (a) pyrene metabolism (7) and prevents N-nitro-N-methylurea-induced mammary tumors in rats (8). It also exhibits various pharmacologic properties such as anti-inflammatory and anticarcinogenic effects. Present effort is to investigate the antidiabetic and antilipemic activity of biochanin A, and its effect on visfatin and other related biochemical parameters in streptozocin-induced diabetic rats. Visfatin is an adipocytokine that is highly expressed in visceral fat; it was originally isolated as a secreted factor that synergizes with IL-7 and stem cell factors to promote the growth of B-cell precursors (9). It is secreted by activated lymphocytes (9) monocytes, and neutrophils (10), stimulates the expression of IL-6 and IL-8 in amniotic cells (11), and prolongs neutrophil survival in clinical sepsis (10). Visfatin was named as such because of its much greater expression in visceral fat than in subcutaneous adipose tissue (12). Along with its insulin-mimetic effects, visfatin was as effective as insulin in inducing hyperglycemia in insulin deficient mice. It acts synergistically with insulin in increasing cellular uptake of glucose, stimulating glucose transfer to the muscle and adipose tissue, and prevents hepatic glucose production. Its insulin-like effects is mediated through direct bounding to and activation of insulin receptors without any change or competition with the insulin (13). 

## 2. Objectives

In our study, we examined the effect of diabetic status on circulating visfatin in comparison with nondiabetic rats. We then assessed the effects of an insulin-mimetic flavonoid, biochanin A, in treatment of rats with type 2 diabetes mellitus and on circulating visfatin levels.

## 3. Patients and Methods

### 3.1. Chemicals and Reagents

Streptozocin and biochanin A were purchased from Sigma-Aldrich. All other chemicals used in this study were of analytical grade obtained from E Merck. Body weight was measured in the morning of the first and the last day of biochanin A administration by Balance: Sartorius TE64 (Germany). Serum concentrations of fasting blood glucose (FBG), triglycerides (TG), total cholesterol, and high-density lipoprotein cholesterol (HDL-c) were measured enzymatically using commercial kits (Pars Azemoon, Tehran, Iran) with the aid of a spectrophotometer (JENWAY 6505, Europe Union). Low-density lipoprotein cholesterol (LDL-c) was calculated by Friedewald formula: LDL-c = Total cholesterol - [HDL-c + (TG/5)] (12). Serum alanine aminotransferase (ALT), aspartate aminotransferase (AST), and alkaline phosphatase (ALP) activities were measured by colorimetric diagnostic kit (ZIESTCHEM Company, Tehran, Iran). Glycosylated hemoglobin (HbA_1_c) was estimated by the method of cation exchange chromatography (Biosystems kit, Barcelona, Spain). The concentration of serum visfatin was determined by commercial enzyme immunoassay Kits (Bioassay technology laboratory, Shanghai, China) with the aid of an ELISA plate reader (ELx800TM, Bio-Tek, Winooski, VT, USA).

### 3.2. Animals

Male Wistar rats (weight, 160-180 g) were purchased from the Central Animal House, Tehran University of Medical Sciences, and were maintained in an air-conditioned room with the mean temperature of 25℃ ± 1℃ and humidity of 55% ± 5% with a 12-hour light/12-hour dark cycle. All the rats were provided with commercially available rat normal pellet diet, which contained carbohydrate 60% (w/w), fat 2% (w/w), protein 17.5% (w/w), and fiber 8% (w/w), and water ad libitum. Institutional Animals’ Ethics Committee of Arak University of Medical Sciences, Arak, Iran, approved the study protocols.

### 3.3. Induction and Selection of Diabetic Rats

After an overnight fasting, 35 mg/kg of freshly prepared streptozocin (in citrate buffer with pH 4.5) was immediately injected intraperitoneally (IP) to induce diabetes. The respective control rats were given vehicle citrate buffer. The FBG was measured three days after streptozocin or vehicle injection. The rats with the FBG > 126 mg/dL were considered diabetic and selected for further studies.

### 3.4. Experimental Design

In this experimental study, 24 male Wistar rats were randomly allocated (simple randomization) to four groups (six rats in each group). One group was randomly selected and assigned as control group (group 1) and diabetes was induced in three other groups by administration of streptozocin (35 mg/kg) IP. Biochanin A was suspended in 0.5% DMSO and administrated orally once a day in the morning for 30 days. The studied groups and attributed treatments were as follow:

Group1: control (0.5% DMSO)
Group 2: diabetic control (0.5% DMSO)
Group 3: diabetic + biochanin A (10 mg/kg/day)
Group 4: diabetic + biochanin A (15 mg/kg/day)

The initial well as final body weight and FBG of all groups were recorded after 30 days in fasting condition. The animals were anesthetized using IP ketamine (75 mg/kg) and xylazine (10 mg/kg). Blood sample was collected by cardiac puncture and serum was separated immediately.

### 3.5. Histopathologic Analysis

On day 30, the pancreas tissues (five samples from each group) were removed and after washing with normal saline, they were stored in 10% formalin. The tissues embedded in paraffin and sectioned (Leica, Wetzlar, Germany), stained with hematoxylin and eosin, and subsequently examined under light microscope (Olympous, Tokyo, Japan).

### 3.6. Statistical Analysis

All the data were expressed as mean ± standard error of three replicates for six rats in each group. Statistical analysis was performed using SPSS (version 19, SPSS Inc., Chicago, IL, USA). Normal assumption was examined using one-sample Kolmogorov-Smirnov test. One-way ANOVA was applied for determining differences between results of the studied groups. Post hoc test was used to compare the data. P values < 0.05 were considered statistically significant.

## 4. Results

Table 1 presents the effect of biochanin A on changes in FBG levels, body weight, and HbA_1_c in diabetic and control rats. Serum glucose level was measured in normal and diabetic rats on day zero and 30th day of oral administration of biochanin A. Three days after streptozocin injection, diabetic rats showed significant increase in the FBG in comparison to control group (day zero). Oral administration of biochanin A at doses of 10 mg/kg and 15 mg/kg for 30 days showed highly significant effects on FBG. Biochanin A with lower dose (10 mg/kg) was more effective on reducing FBG in comparison with the higher dose.

Diabetic rats showed decrease in body weight and administration of biochanin A at two different doses for 30 days improved body weight to almost normal limits. Between the two different doses, 10 mg/kg was more effective than 15 mg/kg when compared to the untreated diabetic rats. HbA_1_c increased significantly in diabetic rats and after treatment with biochanin A, the values were brought towards normal level. Oral administration of biochanin A at a dose of 10 mg/kg was more effective in decreasing HbA1c. However, the HbA_1_c was not completely reduced in comparison to normal rats.

The effects of biochanin A on lipid profile is shown in Table 2. Serum HDL-c was significantly decreased and the level of TG was significantly increased in diabetic rats in comparison to group 1 rats. Total cholesterol and LDL-c increased in diabetic rats although it was insignificant. Oral administration of 10 mg/kg biochanin A restored the lipid profile to near normal limits. In this dose, total cholesterol, TG, and LDL-c were significantly decreased and the level of HDL-c was significantly increased in treated rats in comparison with the diabetic rats. There were not any significant changes in lipid profile in rats consuming 15-mg/kg biochanin A.

The activities of AST, ALT, and ALP were significantly increased in diabetic rats when compared to control group. Oral administration of biochanin A at a dose of 10 mg/kg was significantly effective and the enzyme activities were brought back to near normal limits in diabetic rats. This dose was more effective than 15 mg/kg was (Table 3). Table 4 shows the effect of biochanin A on changes in visfatin concentration in normal and diabetic rats. Serum visfatin levels were elevated in diabetic rats. The mean serum level of visfatin in groups receiving biochanin A treatment was significantly decreased in comparison to untreated diabetic rats. Biochanin A was more effective at 15 mg/kg than at 10 mg/kg.

**Figure 1.**
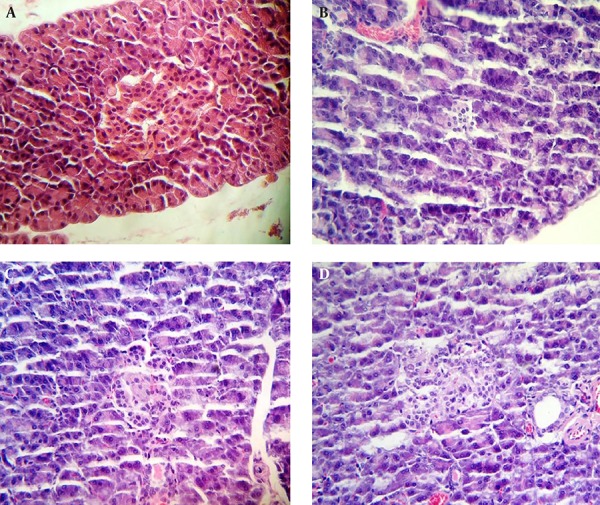
Effect of Biochanin A on Pancreatic Islet Tissue (Hematoxylin and Eosin staining; magnification, × 400) A, Normal rats. B, streptozocin-induced diabetic rats. C, streptozocin-induced diabetic rats treated with 10-mg/kg biochanin A. D, streptozocin-induced diabetic rats treated with 10-mg/kg biochanin A.

The histopathologic assessment of pancreatic section showed that the islets in normal group displayed the complete structure and uniform arrangement (Figure 1A). As shown in Figure 1B, the number and size of pancreatic islets was decreased in diabetic group and sever pancreatic damage and degranulation of beta cells, vacuolation, and invasion of connective tissues were evident; some of them even showed apoptosis or necrosis. After treatment with biochanin A, the structure of islet was improved and histopathologic signs were ameliorated considerably. The cellular morphology of islet showed some degree of recovery. Amelioration and protective effects on pancreatic tissues was more in group 4 (Figure 1 D) than in group 3 (Figure 1 C).

**Table 1.** Effect of Oral Administration of Biochanin A on Fasting Blood Glucose Levels, Body Weight, and Glycosylated Hemoglobin in Studied Rats ^a,b^

Group	FBG (mg/dL)		Body weight (g)		HbA_1_c (%)
	Day 0	Day 30	Day 0	Day 30	
**Normal**	68 ± 3.4	61 ± 3.5	189.3 ± 3.2	210.8 ± 6.5	5.19 ± 0.22
**Diabetic Control**	225 ± 3.8 ^c^	295.1 ± 14.2 ^c^	191.8 ± 4.5	161.5 ± 6.7 ^c^	8.26 ± 0.44 ^b^
**Diabetic + Biochanin A (10 mg/kg)**	215 ± 12.6 ^c^	139.8 ± 9.3 ^c,d^	190.6 ± 3.1	200.5 ± 2.6 ^d^	6.66 ± 0.35 ^d^
**Diabetic + Biochanin A (15 mg/kg)**	221 ± 11.5 ^c^	206 ± 11 ^c,d^	188.6 ± 5.1	195.0 ± 6.3 ^d^	7.11 ± 0.31 ^d^

^a^ Each value is mean ± SD of six rats in each group.

^b^ Abbreviations: FBG, fasting blood glucose; and HbA_1_c, glycosylated hemoglobin.

^c^ P < 0.05 in comparison with normal rats.

^d^ P < 0.05 in comparison with diabetic rats.

**Table 2.** Effect of Oral Administration of Biochanin A on Serum Lipids in Studied Rats ^a,b^

Groups	Total Cholesterol, mg/dL	TG, mg/dL	LDL-c, mg/dL	HDL-c Cholesterol, mg/dL
**Normal**	102.3 ± 9.64	84.33 ± 1.75	49.30 ± 8.4	36.17 ± 2.48
**Diabetic Control**	106.5 ± 4.6	89.50 ± 1.51 ^c^	58.43 ± 5.03	30.17 ± 1.47 ^c^
**Diabetic + Biochanin A (10 mg/kg)**	95.17 ± 4.16 ^d^	75.67 ± 3.55 ^d^	41.36 ± 4.3 ^d^	38.67 ± 0.81 ^d^
**Diabetic + Biochanin A (15 mg/kg)**	111.17 ± 5.8	93.17 ± 4.16	59.70 ± 7.1	32.83 ± 1.94

^a^ Each value is mean ± SD of 6 rats in each group.

^b^ Abbreviations: TG, triglyceride; LDL-c, low-density lipoprotein cholesterol; and HDL-c, high-density lipoprotein.

^c^ P < 0.05 in comparison with normal rats.

^d^ P < 0.05 in comparison with diabetic rats.

**Table 3.** Effect of Oral Administration of Biochanin A on Serum alanine aminotransferase, aspartate aminotransferase, and alkaline phosphatase in Studied Rats ^a,b^

Groups	AST, U/dL	ALT, U/dL	ALP, U/dL
**Normal**	131.33 ± 5.00	52.17 ± 1.83	147.0 ± 6.35
**Diabetic Control**	200.0 ± 6.7 ^c^	98.33 ± 3.93 ^c^	188.0 ± 3.28 ^c^
**Diabetic + Biochanin A (10 mg/kg)**	151.33 ± 5.04 ^d^	67.00 ± 6.29 ^d^	174.0 ± 3.68 ^d^
**Diabetic + Biochanin A (15 mg/kg)**	176.67 ± 7.3 ^d^	78.0 ± 2.09 ^d^	183.6 ± 4.36

^a^ Each value is mean ± SD of six rats in each group.

^b^ Abbreviations: ALT; alanine aminotransferase, AST; aspartate aminotransferase, ALP; alkaline phosphatase.

^c^ P < 0.05 in comparison with normal rats.

^d^ P < 0.05 in comparison with diabetic rats.

**Table 4.** Effect of Oral Administration of Biochanin A on Serum Visfatin Concentration in Studied Rats ^a^

Groups	Serum Visfatin, mg/dL
**Normal**	188.63 ± 23.6
**Diabetic Control**	302.17 ± 19.4 ^b^
**Diabetic + Biochanin A (10 mg/kg)**	249.00 ± 35.49 ^c^
**Diabetic + Biochanin A (15 mg/kg)**	161.33 ± 13.07 ^c^

^a^ Each value is mean ± SD of 6 rats in each group.

^b^ P < 0.05 in comparison with normal rats.

^c^ P < 0.05 in comparison with diabetic rats.

## 5. Discussion

In the present study, we observed significant effect of oral administration of biochanin A on FBG, body weight, HbA_1_c, lipid profile, liver enzyme activities, and serum visfatin concentration in streptozocin-induced diabetic rats. Although high dose of streptozocin severely impairs insulin secretion mimicking type 1 diabetes, low dose of streptozocin destroys some population of pancreatic beta cells and has been known to induce a mild impairment of insulin secretion, which is similar to the feature of the later stage of type 2 diabetes (13). There was no significant variation in plasma insulin concentrations between diabetic and normal rats while FBG was significantly higher in diabetic rats; therefore, insulin resistance might have been developed in these animals. We used an animal model that would closely reflect the natural history and metabolic characteristics of human and it is further sensitive to pharmacological testing.

Our results showed that the increased levels of glucose in streptozocin-induced diabetic rats were significantly lowered, but the level of blood glucose in treatment groups was still higher than the normal group. Reduction in FBG by the administration of biochanin A suggested that it might increase the insulin secretion, which in turn raises glucose uptake by tissues. Biochanin A treatment effectually enhanced insulin secretion and a better glycemic control as well as antioxidant protection (14).

Brahmachari reported that flavonoids are naturally-occurring phenolic compounds with a broad range of biologic activities and the beneficial effects of them have been studied in relation to diabetes through inhibition of intestinal alpha-glycosidase enzyme, their capacity to avoid glucose absorption, and/or to improve glucose tolerance (15). Biochanin A, a soy isoflavone, specifically has antidiabetic activity. The possible mechanism by which the biochanin A brings about hypoglycemic effect may be increasing the insulin level because of its protective effect on pancreatic beta cells and stimulation of insulin secretion from the remaining beta cells.

The streptozocin-induced diabetic rats significantly lost weight in comparison to control rats (16). Sever weight loss is a characteristic condition in diabetes; several studies have also reported a significant decrease in body weight of streptozocin-induced diabetic rats (17) owing to defect in glucose metabolism and excessive breakdown of tissue proteins (18). The present study, showed that after 30 days of oral administration of biochanin A, the rat health status were to some extent improved along with a good spirits. Simultaneously, the body weight significantly and gradually improved. Increase in the body weight of streptozocin-induced diabetic rats might be due to an improvement in insulin secretion and glycemic control. Increased insulin levels may contribute to the reduced hyperglycemia and improved body weight in treated diabetic rats (19). HbA_1_c levels were found to be significantly elevated in streptozocin-induced diabetic rats in comparison to the normal rats. Treatment with biochanin A decreased hyperglycemia and therefore, exhibited a significant reduction in the levels of HbA_1_c.

Serum TG levels was significantly higher in the diabetic untreated rats in comparison to normal rats; on the other hand, although total cholesterol and LDL-c levels were higher, the difference was in significant. In addition, the levels of HDL-c were significantly lower in the former group. The abnormally high concentrations of serum lipids in the diabetic subjects are mainly due to the increase in the mobilization of free fatty acids from the peripheral fat depots since insulin inhibits the hormone-sensitive lipase. Insulin deficiency or insulin resistance may be responsible for dyslipidemia because insulin has an inhibitory action on HMG-CoA reductase, a key rate-limiting enzyme responsible for the metabolism of cholesterol-rich LDL-c particle (20). An abnormality in lipid profile is one of the most common complications in diabetes and is associated with an increased risk of coronary heart disease (21, 22). Since lipid abnormalities accompanying with premature atherosclerosis are the major causes of cardiovascular disease in patients with diabetes, in addition to glycemic control, ideal treatment for diabetes, should have a favorable effect on lipid profile (23). After 30 days treatment with biochanin A (10 mg/kg), a significant reduction in serum TG, total cholesterol, and LDL-c and a significant increase in the HDL-c level was observed. The glucose lowering action of the biochanin A might be due to consequent improved lipid metabolism apart from direct interaction with glucose homeostasis. The TG-lowering property could indirectly contribute to the overall antihyperglycemic activity through glucose-fatty acid cycle mechanism (24). According to this mechanism, increased supply of plasma TG per se could constitute a source of increased fatty acid availability and oxidation that can impair insulin action and hence, glucose metabolism and utilization that leads to development of hyperglycemia. Therefore, the reduction of TG following treatment with biochanin A would also facilitate the glucose oxidation and utilization and subsequently, the reduction of serum glucose.

The liver is regarded as the central organ of metabolism in the body with an important role in glucose and lipid metabolism (25). AST, ALT, and ALP are considered as liver toxicity markers and are used in the evaluation of hepatic disorders (26). Our result showed that serum AST, ALT, and ALP were increased in streptozocin-induced diabetic rats in comparison with normal rats. The increase in the activities of these serum enzymes indicated that diabetes might induce hepatic dysfunction and reflect active liver damage. Therefore, the increase in AST, ALT, and ALP activity may be mainly due to the leakage of these enzymes from the liver cytosol in to the blood serum, which gives an indication of the hepatotoxic effect of streptozocin (27). Oral administration of biochanin A, especially with a dose of 10 mg/kg, significantly decreased these enzymes in comparison with the control diabetic rats.

Little evidence is available for the effects of biochanin A polyphenols on visfatin expression in diabetic rats. There are reports showing an association between visfatin and metabolic syndrome (28). While elevation in visfatin level is observed in obese subjects, some researchers did not find any differences in this factor between those with metabolic syndrome and healthy controls (28-30). Since visfatin has been known as a proinflammatory cytokine (31), our finding that showed its elevation in diabetic rats were reasonable. Despite these finding about visfatin, its relationship with diabetes and metabolic syndrome still remains to be illuminated.

Our data also showed treatment of diabetic rats with biochanin A could lead to diminished visfatin level. Recent studies have indicated that visfatin has insulin mimetic effects in cultured adipocytes, myocytes, and hepatocytes and can reduce the plasma glucose levels in mice through binding and activating the insulin receptor (31). In addition, it is important to mention that visfatin acts as a phosphoribosyl transferase inside the cell and is involved in salvage pathway of NAD^+^ biosynthesis. This regulation of cellular levels of NAD^+^ affects the NAD^+^/NADH dependent enzymes (32).

According to histopathologic findings, damage in islet with pancreatic beta cells was ameliorated by administration of biochanin A. These findings demonstrated that biochanin A could exert the hypoglycemic and antilipemic effects through the mechanisms that might be associated with repairing pancreatic beta cells in diabetic rats.
